# Factors Associated with the Variation in Drug Prescription of Analgesics in Long-Term Care Facilities: A Systematic Review

**DOI:** 10.3390/jcm14217833

**Published:** 2025-11-04

**Authors:** Rabia Bibi, Mariem Hachani, Alice Masini, Andrea Conti, Giovanni Cangelosi, Sophia Russotto, Francesco Barone-Adesi, Massimiliano Panella

**Affiliations:** 1 Department of Translational Medicine, University of Eastern Piedmont, Via Solaroli 17, 28100 Novara, Italy; 20036124@studenti.uniupo.it (R.B.); andrea.conti@uniupo.it (A.C.); 20015279@studenti.uniupo.it (S.R.); francesco.baroneadesi@uniupo.it (F.B.-A.); massimiliano.panella@uniupo.it (M.P.); 2 Doctoral Program in Food, Health, and Longevity, University of Eastern Piedmont, Via Solaroli 17, 28100 Novara, Italy; 3 Division of Clinical Epidemiologyof Early Cancer Detection, German Cancer Research Center (DKFZ), Im Neuenheimer Feld 581, 69120 Heidelberg, Germany; mariem.hachani@dkfz-heidelberg.de; 4 School of Pharmacy, Experimental Medicine and “Stefani Scuri” Public Health Department, University of Camerino, 62032 Camerino, Italy; giovanni01.cangelosi@unicam.it; 5 Medical Directorate of Hospital Facilities, University Hospital of Alessandria, Via Venezia 16, 15121 Alessandria, Italy

**Keywords:** analgesics, prescription, pain management, older adults, long-term care facilities

## Abstract

**Background/Objectives**: Chronic pain conditions are common among older residents in long-term care facilities (LTCFs), often leading to increased demand for analgesic drugs. Despite this, pain is frequently underdiagnosed and undertreated, especially in individuals with cognitive impairments such as dementia. Both underuse and overuse of analgesics remain a challenge in LTCFs, affecting patient outcomes and quality of life. This systematic review aimed to identify patient- and facility-level factors associated with variation in analgesic use in LTCFs. **Methods**: This review followed PRISMA guidelines. A comprehensive literature search was conducted in PubMed, CINAHL, Cochrane Library, and Scopus for studies published between 2013 and 2024. Eligible studies were focused on adults aged ≥ 60 years in LTCFs and examined patient and facility factors related to analgesic prescription. Risk of bias was assessed independently by two reviewers using STROBE, JBI, and MMAT tools. Disagreements were resolved by a third reviewer. **Results**: A total of 6266 studies were retrieved; 13 papers met the eligibility criteria after screening. Dementia was the most frequently reported factor associated with lower analgesic prescribing, largely due to difficulties in assessing pain and communication barriers. Other patient-related factors included pain severity and comorbidities. Facility-level factors such as staffing levels, staff qualifications, and facility size were also associated with variation in prescribing practices. **Conclusions**: Dementia highly impacts pain management due to assessment and communication challenges. Improved pain assessment tools and staff training are needed to recognize pain in cognitively impaired residents and ensure appropriate analgesic use in LTCFs.

## 1. Introduction

According to the 2020 “Ageing and Health” report by the World Health Organization (WHO), the global population of people aged 80 and above is expected to triple by 2050, reaching 426 million individuals worldwide, substantially increasing the demand for long-term and specialized healthcare [1]. This demographic shift is also expected to lead to a higher prevalence of persistent pain-related conditions such as back and neck pain, osteoarthritis, and refractory pain [1]. In addition, aging is accompanied by chronic pain conditions and mental health disorders (e.g., dementia, depression), both of which can further complicate pain recognition and management in older adults [2,3]. As a result, the demand for receiving special care for pain management in care facilities will rise [4]. Furthermore, in the United States (USA) alone, there is an increased prevalence of the elderly in long-term care facilities (LTCFs); approximately 1.3 million elderly individuals reside in LTCFs, including Nursing Homes (NHs) and assisted living residences [5]. Across the EU (27 countries in 2020), around 3.4 million institutional LTCF beds were available, and approximately 3.6% of people aged 65 and above were actually receiving institutional care [2].

Despite this large and growing population, evidence indicates that residents in LTCFs often receive suboptimal pain management. A recent study reported that up to 80% of residents experience pain, but it is often under-assessed or under-treated [6]. Inadequate pain diagnosis and treatment in nursing homes often lead to inconsistent use of analgesics, which can compromise patient well-being and quality of life. Previous studies have shown that pain symptoms in older adults are often misinterpreted as behavioral disturbance, leading to inappropriate use of psychotropics instead of analgesics [7]. This phenomenon is particularly common in residents with dementia, where behavioral symptoms such as agitation or aggression are often interpreted as cognitive issues rather than as a potential underlying pain [8].

Furthermore, due to challenges in pain assessment among residents, pain is often underdiagnosed. Indeed, pain is the most underreported condition in NHs, which leads to reliance on antipsychotics or sedatives to manage distressed patients [9].

Research suggests that healthcare providers may perceive older adults as less sensitive to pain compared to younger individuals, leading to undermedication, which might contribute to inadequate pain management [10]. Moreover, physicians are often hesitant to prescribe appropriate levels of pain relief due to gaps in knowledge and negative attitudes toward pain [11]. Similarly, studies indicate that nurses in East Asia, as well as those in the USA, Australia, and Finland, display limited knowledge and unfavorable attitudes toward effective pain management practices [12,13]. In fact, this variation, characterized by both overuse and underuse, poses a significant challenge for effective pain management among elderly residents in LTCFs, often leading to unnecessary side effects of unrelieved pain [13]. Therefore, identifying the current best practices and areas in need of improvement is essential. This could contribute to better pain management, reduced adverse drug events, and overall improved quality of life for elderly residents.

Hence, the aim of the present systematic review is to identify and describe patient- and facility-level factors associated with differences in analgesic prescribing practices in long-term care facilities.

## 2. Methods

The present systematic review followed the reporting guidelines and criteria of the Preferred Reporting Items for Systematic Reviews (PRISMA) 2020 Statement [14]. The PRISMA Checklist is available in the Supplementary Materials [see Table S1]. The study protocol is available in the International Prospective Register of Systematic Reviews—PROSPERO (ID: CRD42023422329).

### 2.1. Search Strategy

We conducted a comprehensive search strategy using Medical Subject Headings (MeSH) and keywords in the following electronic databases: PubMed/Medline, Cochrane Library, CINAHL, and Scopus. We searched for the studies conducted from 1 January 2013 to 1 January 2024. We also used a snowball approach in the relevant systematic reviews and manually searched the reference list of included studies to identify and extract any potential articles that may have been missed by the database search. The full search string is provided in Supplementary Table S2.

### 2.2. Inclusion and Exclusion Criteria

We included studies conducted on older adults (≥60 years of age) who received all types of analgesics and resided in LTCFs such as nursing homes, residential care, or skilled nursing facilities. Moreover, we included only studies based on primary data (i.e., cross-sectional studies, cohort studies (prospective and retrospective), experimental, and quasi-experimental studies) that examined analgesic prescription patterns, use, and variation for pain management.

We excluded studies conducted on terminally ill patients receiving special care as we are interested in the contributing factors associated with variation in analgesic use across different LTCFs. We excluded studies conducted in home-based and hospital settings. We included only English-language articles published in the last eleven years (2013–2024) to focus on the most recent and relevant research evolving the pain management in LTCF. For the present systematic review, we define the factors associated with the variability in analgesic use across different LTCFs, which allowed us to identify the diverse contexts and conditions under which the analgesic use may vary. The full PICOST (Participants, Interventions, Comparators, Outcomes, Study design, and Timing) criteria for inclusion and exclusion are presented in Supplementary Table S3.

### 2.3. Study Selection and Data Extraction

Search results from each database were imported into an Excel spreadsheet. Titles and abstracts of retrieved references were screened for relevance by two independent reviewers (R.B. and M.H.). In case of disagreements, a third reviewer (A.M.) cast the deciding vote. In the second stage, two reviewers (R.B. and M.H.) independently reviewed full texts of studies, excluding records not relevant to our research question, and then they extracted the data. These procedures were supervised by a third reviewer (A.M.) in case of disagreement. The following data was extracted: author, year of publication, study design, county of the study, sample characteristics (includes, age, gender, and number of participants), type of prescribed analgesic, facility characteristics (includes, occupancy, staffing level, ownership, staff-related factors, and variation based on geographical location), and patient characteristics (i.e., prevalence of multiple conditions, severity rate, and scale).

Given the heterogeneity of study designs (quantitative, qualitative, and mixed-methods), a narrative synthesis approach was used. We summarized the qualitative findings in both table and narrative texts, complemented by a description to illustrate key observations. Where available, quantitative data (such as means, ranges, and percentages) were also reported. Data from qualitative studies were extracted using predefined categories based on key themes and categories that aligned with our research question. This approach allowed for a structured presentation and interpretation of relevant findings.

### 2.4. Risk of Bias Assessment

Two independent reviewers (R.B. and M.H.) used standardized checklists from the Joanna Briggs Institute (JBI), mixed methods appraisal tool (MMAT), and Strengthening the Reporting of Observational Studies in Epidemiology (STROBE) to evaluate the quality of included studies [15,16]. Since the STROBE checklist does not provide a predefined scoring system, we adopted the cut-off values used in a previous publication: a score of 0–14 represented a high risk of bias, 15–25 represented a medium risk of bias, and 26–33 represented a low risk of bias [17]. We used the JBI checklists for a qualitative study. The following cut-off values were given to the JBI tool to assess the quality of the studies: If studies scored 49% or less, they were considered high risk of bias; studies scoring between 50% and 69% were considered medium risk of bias; and studies with positive responses greater than 70% were regarded as low risk of bias [18,19]. The mixed methods appraisal tool (MMAT) is a validated critical appraisal tool specifically designed to evaluate empirical studies and also mixed-method studies [20]. The tool comprises five methodological criteria according to study design. We used an appraisal that covered three domains: qualitative, quantitative, and the integration of both (i.e., mixed methods logic). According to MMAT guidance, the overall quality of a mixed methods study is determined by the lowest score among its three components, as the study’s rigor cannot exceed that of its weakest part. Although the MMAT discourages calculating a composite score, a descriptive scoring system was applied for clarity and comparison; i.e., each criterion that was met was awarded one point (out of five).

## 3. Results

### 3.1. Identification of Studies

**Figure 1:**
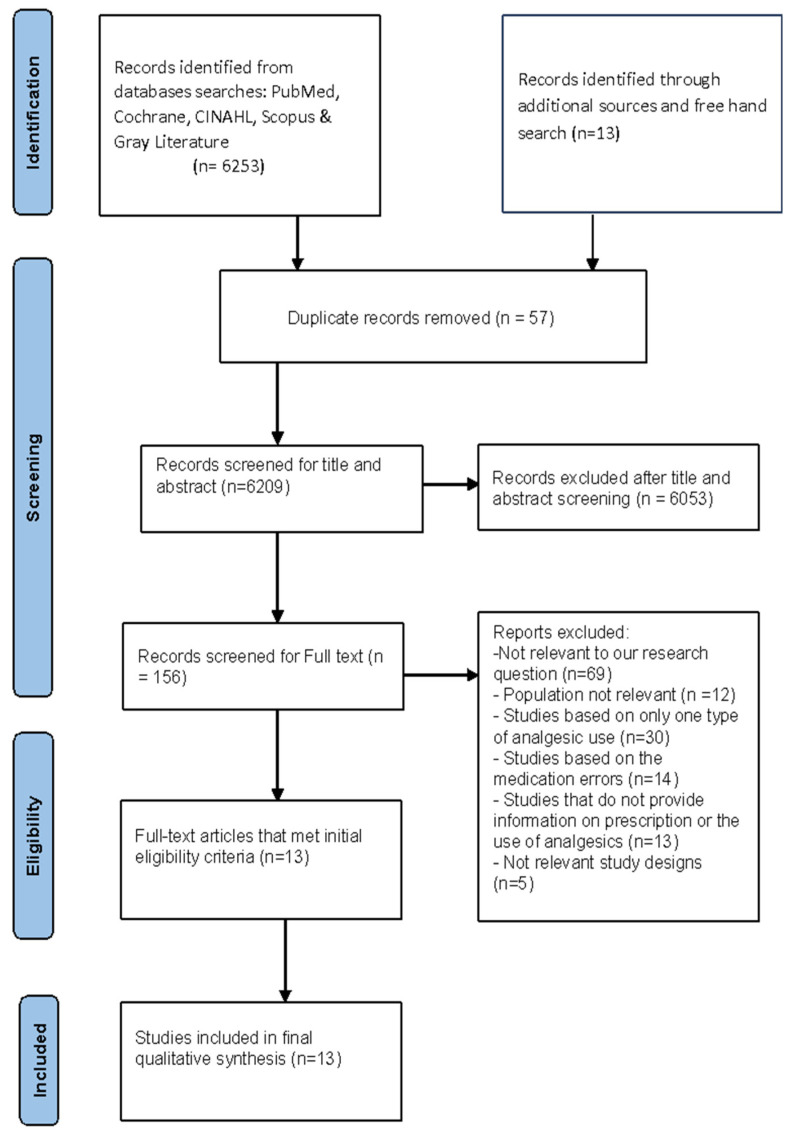
PRISMA flowchart for the article selection.

A total of 6253 studies were retrieved from the electronic databases search. Additionally, 13 records were identified through additional sources and a hand search. After deleting the duplicate, 6053 papers were screened for title and abstract. Therefore, 143 records were screened, and 130 full texts were excluded. Finally, 13 studies were included in our review. The complete PRISMA flowchart is shown in Figure 1.

### 3.2. Study Characteristics

Table 1 shows the characteristics of the included studies. Of the thirteen studies, nine were cross-sectional studies [21,22,23,24,25,26,27,28,29], two were cohort studies [30,31], one was a qualitative study [32], and one used mixed methods [33]. Based on the country of origin, the included studies are as follows: United States (*n* = 4) [21,27,30,32]; Canada (*n* = 2) [28,31]; Norway (*n* = 1) [29]; Australia (*n* = 1) [26]; Denmark (*n* = 1) [23]; France (*n* = 1) [22]; Northern Ireland (*n* = 1) [33]; and Netherlands (*n* = 1) [24]. In addition, there was one study based in seven EU countries (the Czech Republic, England, Finland, France, Germany, Italy, the Netherlands) [25].

The studies showed notable diversity based on sample size, mean age of participants, and type of analgesics prescribed. Specifically, sample sizes ranged from 98 to 1,156,875 residents [26,27], while the average age varied from 60.0 years to 87.0 years [22,24]. Female representation also varied greatly, with the highest proportion reported by Fain et al. (83.9%) and the lowest by Mehta et al. (20.9%) [21,30].

### 3.3. Prevalence of Painkillers

Across the studies, the prevalence of analgesic use in LTCFs varied widely by country and by sample characteristics. Opioid use was generally high, particularly in North America. Iacono et al. reported the highest prevalence, with approximately 68.7% of Canadian nursing home residents being prescribed opioids [28]. Another study observed 64.5% opioid use in residents, predominantly female U.S. sample (83.9% female), whereas Lapane et al. reported 46.4% opioid use and 13.2% non-opioid analgesic use among 48.9% male [21,27]. However, Mehta et al. (2021) reported usage rates declining from 14.1% in 2014 to 11.4% in 2018 in a large U.S. cohort (*n* = 3,245,714) [30]. In Europe, use of analgesics differed greatly by region. Lukas et al. (2013), in a multi-country study covering seven European countries and Israel (*n* = 4156), found that 50% of residents were prescribed paracetamol and 12% received opioid-containing combinations [25]. Jensen-Dahm et al. reported a 41% opioid use rate in Danish nursing homes, while Barry et al. reported the lowest opioid use of 4.8% and the highest use of paracetamol (69%) in Northern Ireland LTCFs. In France, the overall analgesic rate was 46.8%, with opioids at 11.4% and NSAIDs at 2.9% [23,33]. In Norway, Sandvik et al. showed a substantial increase in paracetamol from 22.7% up to 48.4% in 2011, prescribing over 12 years in Norway. Notably, they also demonstrated fluctuations in opioid use, with a marked increase from 10.9% in 2000 to 23.8% in 2011, while NSAID use, in contrast, declined sharply over time from 6.8% (2000) to 3.2% (2011) [17].

### 3.4. Patient and Facility Characteristics

**Figure 2:**
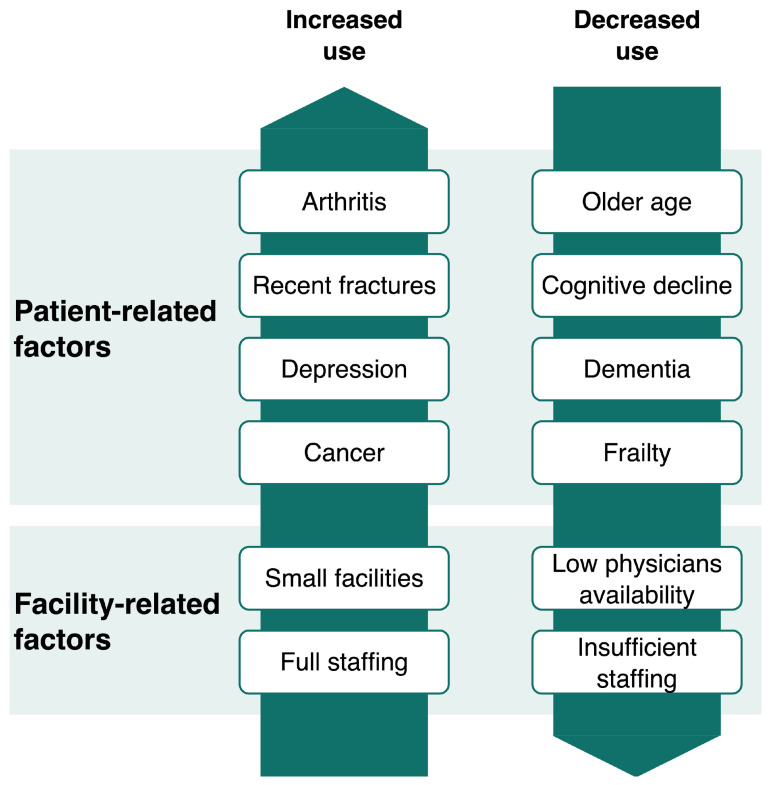
Summary of patient- and facility-related factors associated with analgesic use.

Table 2 summarizes patient and facility factors associated with the variation in analgesic prescription rate in LTCFs. Figure 2 provides a visual summary of these factors.

#### 3.4.1. Patient-Related Factors

Among the patient-related factors, four domains were observed: type of disease, comorbidities, pain severity, and patient demographic characteristics.

##### Diseases Associated with Analgesic Prescribing

Certain diseases were consistently associated with variability in analgesic prescribing. Most studies reported dementia as the most frequent diagnosis associated with lower analgesic prevalence, ranging from 42% to 87% [28,29,31,33]. Cancer was the second most frequently reported disease, with four studies—Jensen-Dahm et al. (15.8%), Veal et al. (11%), Lukas et al. (13%), and Barreto et al. (12.7%)—reporting a positive association with opioid use, including among female patients, as especially found by Veal et al. and Lukas et al. [22,23,26]. Other conditions, such as arthritis, fractures, musculoskeletal pain, cognitive impairment, and frailty, were less consistently reported; however, these conditions were more associated with the increased use of opioids. A decline in cognitive function and depression appeared in multiple studies—Barreto et al. (34.2%), Veal et al. (37.7%), Iacono et al. (26.9%), and Lapane et al. (41.6%)—and were generally associated with increased analgesic use, in particular opioid prescriptions [22,27,28]. While higher comorbidity scores were positively associated with analgesic use, with prevalence rising from 23.3% for scores 0–4 to 27.9% for 5–9 and 48.9% for 10 [31].

##### Pain Severity

A total of six studies reported pain severity and its association with analgesic use [22,25,26,27,30,33]. Lukas et al. and Veal et al. found that higher pain severity and musculoskeletal pain were linked to increased analgesic use [25,26]. Mehta et al. observed that individuals reporting mild or frequent pain (28.3%) were more likely to use opioids chronically, compared to those with moderate or severe pain (18.1%) [30]. In contrast, Barreto et al. reported that 23.4% of residents documented pain complaints, but no association with analgesic use was found. However, Barry et al. reported that unfamiliarity with pain assessment documentation is associated with lower analgesic use [21,22].

#### 3.4.2. Facility Characteristics

Several facility factors are observed, which are divided into the following domains: staffing levels, ownership, and facility size.

##### Staffing Levels

Five studies reported that staffing levels and qualifications are associated with high analgesic use in LTCFs. Barry et al. reported that an increased number of care assistants (*n* = 16), with 75% working full-time and 43.8% having qualifications, was associated with a higher prevalence of pain medication prescriptions [33]. Fain et al. reported that more staff hours per 42 residents (4.0–4.5 h/resident/day) was associated with a high prevalence of analgesic use [21]. Corazzini et al. observed that inadequate staffing levels are associated with low prescription levels [32]. Meanwhile, Mehta et al. reported that an increase in opioid prescriptions by nurse practitioners and physician assistants leads to increased variation in prescribing practices [30]. Lukas et al. reported that low-to-moderate physician availability (staff mix availability) was negatively associated with analgesic treatments [23].

##### Ownership

Two studies reported the association of ownership with analgesic use in LTCFs [23,31]. However, only one study explicitly mentioned its association with increased opioid prescribing [31].

##### Facility Size

Facility size varied across the studies and showed important associations with analgesic prescribing [30,31]. Rochon et al. reported that the majority of patients residing in large LTCFs (72.4%) received special care for residents with high comorbidity and cognitive impairment, which correlated with higher opioid use [31]. Mehta et al. reported differences by facility size, indicating that smaller nursing homes (32.8%) had higher chronic opioid use compared to larger facilities (11%) [25].

### 3.5. Risk of Bias Assessments

Table 3 represents the quality of included studies. One study was assessed using the JBI tool and one using the MMAT; these were rated as having a low and medium risk of bias, respectively [32,33]. Corazzini et al. did not provide sufficient information regarding the influence of the researchers on certain aspects of the study [32]. Barry et al. lacked a detailed descriptive quantitative analysis and failed to address inconsistencies between the quantitative and qualitative findings [33]. Based on the STROBE checklist, eight studies were regarded as having a medium risk of bias [22,25,26,27,28,29,30,31]. Common reasons for the medium ratings included lack of an explanation for how the study size was determined, failure to address potential sources of bias (particularly Items 9 and 10), and the absence of detailed descriptive information in the results (Item 13). The individual scoring of each study according to the JBI, MMAT, and STROBE checklists is provided in Supplementary Tables S4a,b and S5a,b.

## 4. Discussion

This review identified wide variability in analgesic prescribing across LTCFs, primarily shaped by both patient- and facility-level characteristics. While the evidence was predominantly descriptive, certain trends emerged consistently: analgesics, particularly opioids, are often used to manage chronic and acute pain, yet their prescribing practices vary widely, raising concerns about both under-treatment and increased use [34]. By examining the broader care environment, including facility characteristics such as staffing levels, ownership, and size, along with individual patient factors such as type of diseases, comorbidities, and pain severity, we aimed to identify key drivers of analgesic use and potential areas for targeted intervention. Understanding these variations is essential for developing standardized care and improving pain management for the older population in LTCFs.

### 4.1. Types of Diseases

Dementia emerged as the most prevalent condition and, therefore, can be considered a typical characteristic of this population. However, the more relevant aspect is the inconsistency in its reported association with analgesic use. While previous evidence has linked dementia to an increased use of analgesics in LTCFs [35], our findings indicate an inverse relation, where dementia has been associated with low analgesic usage [22,29]. Consistent with our findings, Tan et al. observed that there is an increasing tendency for analgesic prescription and administration rates to be lower in dementia patients due to challenges in pain assessment and management for this population [3]. Corbett et al. observed that pain is underreported in dementia patients, which leads to inadequate pain treatment, ultimately leading to increased behavioral and psychological symptoms [36]. This gap largely reflects that current pain management practices may not be appropriately tailored for dementia patients, raising concerns about equity in care for dementia patients. In regard to arthritis and fracture history, an overall increasing trend was observed in both opioids and non-opioid use, particularly among residents with musculoskeletal conditions [37,38].

Cancer, particularly in its more aggressive forms, is typically associated with significant pain, with a strong association with analgesic use. This may suggest that pain management in cancer patients within LTCFs is influenced by the cumulative comorbidities, which outweigh the cancer diagnoses for such residents [34].

### 4.2. Staffing Levels

The interaction between staffing adequacy and patient factors appears pivotal. For instance, staff instability, such as high turnover and a low staff mix, including low physician availability, was negatively associated with analgesic treatment, potentially reflecting reduced clinical oversight and delayed responses to residents’ pain needs [2]. Additionally, the absence of registered nurses and overall inadequate staffing have been associated with inappropriate prescribing patterns, as observed by Fagerström et al., who emphasized the important role of non-physician prescribers such as nurse practitioners and physician assistants in improving pain management outcomes through more frequent assessments and personalized care [3]. Similarly, Petyaeva et al. reported that staff training and confidence were key factors in enhancing pain management, further highlighting the influence of staff competence on resident outcomes [4]. Taken together, these findings suggest that both under-prescription and over-prescription of analgesics may result from inadequate staffing, leading to inaccurate assessments and suboptimal decision-making. Conversely, in understaffed LTCFs, reduced clinical attention likely amplifies the effects of dementia and comorbidity on the undertreatment of patients. This reinforces that organizational structure does not act in isolation; it is a compounded effect of organizational-level and patient-level barriers. However, it is important to note that the available literature on staff-related factors and their direct association with analgesic use remains sparse.

### 4.3. Comorbidities

We observed that, rather than isolated disease categories, it is the cumulative burden of illness that predicts analgesic use, supporting a multidimensional approach to pain management. This is supported by the findings of Rochon et al., where analgesic use increased proportionally with higher comorbidity scores, suggesting that the cumulative burden of illness, rather than the presence of any single condition, may be a more critical driver of opioid use [31]. Similarly, Grant et al.’s findings highlighted the same issue, indicating that debilitating conditions often require pharmacological interventions [39]. Indeed, Fain et al. reported that declining cognitive function can hinder accurate self-reporting of pain, potentially leading to under-recognition or underreporting, or perhaps the prescribers take a cautious approach due to concerns about side effects and adverse outcomes [40,41]. Therefore, future research should stratify by comorbidity index rather than single diagnoses to better quantify prescribing inequities.

### 4.4. Pain Severity

Pain severity is usually measured by standardized indices in the older population, particularly for those with musculoskeletal conditions such as arthritis and other degenerative joint diseases, which are major sources of chronic pain [42,43,44]. Serhal et al. reported that accurately assessing pain severity among patients with dementia can be challenging; however, it is essential for providing appropriate pain management [45]. El Tallaway et al. suggested that the reasons for poor pain assessment and management involve communication barriers, limited expertise of healthcare workers, misconceptions, particularly regarding the pharmacological treatment of pain, and misinterpretation of patient behavior [46,47]. Therefore, we believe that the use of validated pain assessment tools can be helpful for caregivers and healthcare providers to identify and quantify pain levels accurately, ensuring that patients with dementia and other challenges can receive adequate analgesia.

### 4.5. Patient Demographics

The female gender was associated with a higher prevalence of analgesic use. Zacroff et al. explained that women are more likely to report pain and are more frequently prescribed analgesics compared to men [48]. There are several factors involved in this; for example, women are more likely to experience pain differently due to biological and hormonal differences, potentially leading to a higher prevalence of conditions associated with chronic pain, such as arthritis and osteoporosis [49,50]. However, our findings did not confirm whether these specific conditions were directly linked to increased opioid use in females. Another important factor is advanced age, which was linked to lower opioid use because of assessing pain in elderly patients [27,38]. Since women are already highly prevalent in nursing homes, it is difficult to establish a clear causal relationship between gender and analgesic use. Instead, demographic effects should be interpreted within a broader facility and health context of women rather than as independent determinants.

### 4.6. Strengths and Limitations

When contrasted with prior reviews, our synthesis expands on earlier evidence by explicitly linking patient factors with organizational capacity. While previous studies described each domain separately, this review highlights how both patient-level vulnerabilities (dementia, frailty, pain underreporting) and organizational constraints (staff shortages, ownership structure) converge to produce the observed prescribing variability. Moreover, by including a diverse range of studies, the review also highlights the relation between analgesic use and various clinical conditions, as well as organizational aspects such as staffing levels and staff training, which helped to draw a conclusion on how the pain is managed in LTCFs among elderly residents.

However, our review has some limitations. First, the studies included were mainly of medium quality, which could have affected our results. In this regard, we observed fewer qualitative or survey-based studies. Although qualitative studies are often smaller in scale and less generalizable, their absence limits our understanding of the lived experiences and perspectives of residents and care staff, particularly regarding the challenges in cognitive impairment or dementia patients. Moreover, several studies did not use validated pain assessment tools, which could contribute to under- or misreporting of pain and analgesic use. It is also important to note that the methodological and population heterogeneity among the included studies limits the comparability and precludes meta-analysis. While valuable for comprehensiveness, the different study designs, dominated by cross-sectional studies (low level of evidence), may reduce the strength of causal inference. Consequently, the findings should be interpreted as indicative trends rather than conclusive evidence. In addition, there is limited research on how staff-related factors, such as training and staffing levels, directly influence analgesic prescribing, which indicates a need for further investigation into these complex dynamics within long-term care settings.

## 5. Conclusions

Our review showed that several factors have the potential to influence the prescription of analgesics for older people in LTCFs. Dementia significantly affects pain management and analgesic prescription due to difficulties in pain assessment and communication. There is a need to develop better standardized pain management protocols, in particular a pain assessment tool specifically designed for cognitively impaired individuals, to ensure equitable analgesic use. Policymakers and administrators should invest in staff training programs focused on non-verbal pain recognition, safe opioid use, and individualized care planning, especially training in consistent use of validated pain assessment tools, further increasing the staff-to-resident ratios, and ensuring multidisciplinary collaborations.

These measures will ensure that all residents, regardless of cognitive status, demographic characteristics, or pain severity, receive appropriate and timely pain management interventions, improving their overall quality of life. Finally, future research should prioritize high-quality, comparable studies using validated assessment tools and harmonized outcome measures to enable cross-country benchmarking. Although this review provides valuable insights into prescribing patterns, most of the included studies were observational and of medium methodological quality. Therefore, the overall evidence should be interpreted with caution, and further high-quality research is needed to strengthen the evidence base on analgesic prescribing practices in long-term care facilities.

## Figures and Tables

**Table 1:** Characteristics of the included studies.

Authors	Country and Study Design	Study Period	Sample Characteristics	Type of Analgesics	Prescription/Use of Analgesics (%)
Barry et al., 2015 [33]	Northern Ireland, mixed methods	2 years	*n* = 42 Female = 57.1% Age = 82.1	Paracetamol, Opioids	Paracetamol = 69%; Opioids = 4.8%
Fain et al., 2017 [21]	United States, cross-sectional	1 year	*n* = 18,526 Female = 83.9% Age = 80	Opioids, NSAIDs	Opioids = 64.5%; NSAIDs = 2.6%
Barreto et al., 2013 [22]	France, cross-sectional	1 year and 8 months	*n* = 6275 Female = 73.7% Age = 87	NSAIDs, Opioids	NSAIDs = 2.9%; Opioids = 11.4%
Jensen-Dahm et al., 2015 [23]	Denmark, cross-sectional	1 year	*n* = 42,291 Female = 71.4% Age = 86.6	Opioids	Opioids = 41%
Martens et al., 2018 [24]	Netherlands, cross-sectional	Not reported	*n* = 142 Female = 75% Age = 60	Opioids	Oxycodone = 61%; Fentanyl = 56%
Mehta et al., 2021 [30]	United States, cohort	4 years	*n* = 3,245,714 Female = 20.9% Age = 83	Opioids	Chronic opioid use: 2014 = 14.1%; 2018 = 11.4%
Lukas et al., 2013 [25]	7 EU countries + Israel, cross-sectional	Not reported	*n* = 4156 Female = 75.6% Age = 83.9	Paracetamol, NSAIDs, Opioids, Metamizole	Paracetamol = 50%; Metamizole = 12%; Tramadol = 9%; Morphine = 7%; Oxycodone = 6%; Fentanyl = 6%; Diclofenac = 4%; Ibuprofen = 2%; Buprenorphine = 2%
Veal et al., 2015 [26]	Australia, cross-sectional	2 years	*n* = 4335 Female = 69.2% Age = 75	Opioids, Fentanyl Patch, Tramadol, NSAIDs	Almost 91% prescribed analgesics: Opioids = 31.8%; Fentanyl = 34%; Tramadol = 4.1%; NSAIDs = 20.8%
Lapane et al., 2020 [27]	United States, cross-sectional	5 years	*n* = 180,780 Male = 48.9% Age = 67	Opioids, Non-opioid Analgesics	Opioids = 46.4%; Non-opioids = 13.2%
Corazzini et al., 2013 [32]	United States, qualitative	Not reported	Not reported	All types of Painkillers	Not reported
Iacono et al., 2022 [28]	Canada, cross-sectional	1 year	*n* = 75,020 Female = 70% Age = 85.1	Opioids	Opioids = 68.7%; Codeine = 16.8%; Oxycodone = 6.8%; Morphine = 5.3%; Fentanyl = 2.6%
Rochon et al., 2023 [31]	Canada, cohort	2 years	*n* = 26,592 Female = 73.3% Age = 66–95+	Opioids, Gabapentinoids, Benzodiazepines	Opioids + Gabapentinoids = 25.4%; Opioids + Benzodiazepines = 4.3%
Sandvik et al., 2016 [29]	Norway, cross-sectional	12 years (2000–2011)	*n* = 1926 Female = 73% Age = 85.6	Paracetamol, NSAIDs, Opioids	2000 Paracetamol = 22.7%, NSAIDs = 6.8%, Opioids = 10.9% 2004 Paracetamol = 35.7%, NSAIDs = 9.1%, Opioids = 9.8% 2009 Paracetamol = 42.7%, NSAIDs = 3.2%, Opioids = 25.1% 2011 Paracetamol = 48.4%, NSAIDs = 3.2%, Opioids = 23.8%

Unless otherwise indicated, data is presented as follows: *n*, total number of residents in LTCFs, and gender in percentage. Age is defined as mean age (Average). Abbreviations: LTCFs, long-term care facilities; NSAIDs, non-steroidal anti-inflammatory drugs. Source: Authors’ elaboration based on included studies.

**Table 2:** Patients and facilities characteristics.

Author	Facility Factors	Patient Factors	Prevalence	Main Findings
Barry et al., 2015 [33]	Staffing level: 16 nurses/care assistants (75% full-time, 43.8% qualified)	Dementia: 85.7%	Dementia patient: high prevalence	Residents on antipsychotics are more likely to receive analgesics. Pain mismanagement and unfamiliarity with pain assessment tools in dementia patients.
Fain et al., 2017 [21]	Staff hours per resident: 4.0–4.5 h/day	Cognitive impairment: 74.4%	Older age: low prevalence	Increased staff hours are linked to higher analgesic prevalence. Older age and severe cognitive impairment are linked to lower analgesic prevalence.
Barreto et al., 2013 [22]	Not reported	Dementia: 53.4%, pain: 23.4%, cancer: 12.7%, depression: 34.2%	Dementia patient: low prevalence	Dementia patients were less likely to receive analgesics due to lack of pain assessment records. Systematic evaluation would reduce misuse.
Jensen-Dahm et al., 2015 [23]	Not reported	Cancer: 15.8%, arthritis: 21%, recent fracture: 8.9%	Cancer and arthritis patients: high prevalence	Residents with comorbidities such as cancer, arthritis, and recent fractures had increased opioid use.
Martens et al., 2018 [24]	83% physicians, 16% physician trainees	Older patients: high prevalence	Opioid prevalence: high (oxycodone = 61%)	Opioid prescribing is influenced by physician experience, with minimal use of guidelines.
Mehta et al., 2021 [30]	Chronic opioid use in nursing home stays, for-profit ownership (66.4%), facility size: small = 32.8%, large = 11%	Pain: mild/frequent = 28.3%, moderate–severe = 18.1%, age: older = low prevalence	Pain severity: high prevalence	Increased opioid use is associated with nurse/physician prescribing, longer nursing home stays, and for-profit facility ownership.
Lukas et al., 2013 [25]	Staff mix (LN:NA HPRD) *: Germany = 100%, England = 94%, Czech Republic = 83%	Dementia: 45.9% fractures: 5.7%, cancer: 13%	Cancer and dementia patients: high prevalence	Female gender, cancer, and pain severity are positively associated with higher analgesic use. Low physician availability is linked to higher PRN ‘as-needed’ prescriptions.
Veal et al., 2015 [26]	Not reported	Musculoskeletal pain: 53.6%, cancer: 11%, osteoporosis: 22.9%, depression/anxiety: 37.7%, dementia: 18.5%	Dementia patients: low prevalence	Higher opioid use among females, especially those with cancer, depression, fractures, or musculoskeletal pain.
Lapane et al., 2020 [27]	Not reported	Mildly impaired cognition: 27.9%, moderately/severely impaired cognition: 26.6%, Alzheimer’s/dementia: 27.6%, depression: 41.6%	Pain severity: high prevalence	Age and cognitive impairment are inversely related to analgesic use. Older age and multiple mental conditions increase the need for pain management.
Corazzini et al., 2013 [32]	Private: 4, for-profit: 6	Not reported	Inadequate staffing: low prevalence	Lack of registered nurses is associated with inadequate care and low analgesic prescription. Focus on optimizing staff collaboration.
Iacono et al., 2022 [28]	Not reported	Frailty: 58.7%, moderate–severe impairment: 47.2%, depression: 26.9%, dementia: 65.8%	Severe pain: high prevalence	Residents with frailty, severe pain, and depression are more likely to receive opioids. Dementia patients had comparatively lower opioid prescription rates.
Rochon et al., 2023 [31]	Small LTCFs: 0.7%, medium LTCFs: 21.5%, large LTCFs: 72.4%, urban: 80.7%, rural: 14%	Comorbidity Score: 0–4 = 23.3%, Alzheimer’s Disease: 13%, Dementia: 42%	Cognitive impairment: high prevalence	High comorbidity and concurrent therapy are positively associated with opioid use. Opioid deprescribing linked to younger age and high comorbidity.
Sandvik et al., 2016 [29]	54 municipalities, special care units (2000–2011)	Female: 70% (2000), 73% (2004), 75% (2009), 71% (2011), dementia: 76–87%	Female Patients: High Prevalence	Female patients and those aged 81–90 had higher analgesic use. Dementia patients had lower analgesic prescriptions, but usage increased from 2000 to 2011.

Abbreviations: LTCFs, long-term care facilities; PRN, pro re nata; LN, licensed nurse; NA, nursing assistant; HPRD, hours per resident per day. * Staff mix (LN:NA HPRD): ratio of LN to NA, expressed as a percentage of total direct care hours per resident per day. Source: authors’ elaboration based on included studies.

**Table 3:** Risk-of-bias assessment of observational studies using STROBE Checklist, JBI tool, and MMAT.

Serial No.	Study	Study Design	Assessment Tool	Risk of Bias	Risk of Bias Score
1	Fain et al., 2017 [21]	Cross-sectional	STROBE	Low	26/33
2	Barreto et al., 2013 [22]	Cross-sectional	STROBE	Medium	25/33
3	Jensen-Dahm et al., 2015 [23]	Cross-sectional	STROBE	Low	26/33
4	Martens et al., 2018 [24]	Cross-sectional	STROBE	High	13.5/33
5	Mehta et al., 2021 [30]	Cohort	STROBE	Medium	17/33
6	Lukas et al., 2013 [25]	Cross-sectional	STROBE	Medium	23/33
7	Veal et al., 2015 [26]	Cross-sectional	STROBE	Medium	20/33
8	Lapane et al., 2020 [27]	Cross-sectional	STROBE	Medium	18/33
9	Iacono et al., 2022 [28]	Cross-sectional	STROBE	Medium	19/33
10	Rochon et al., 2023 [31]	Cohort	STROBE	Medium	20.5/33
11	Sandvik et al., 2016 [29]	Cross-sectional	STROBE	Medium	15/33
12	Corazzini et al., 2013 [32]	Qualitative	JBI	Low	70%
13	Barry et al., 2015 [33]	Mixed methods	MMAT	Medium	60%

JBI, Joanna Briggs Institute; STROBE, Strengthening the Reporting of Observational Studies in Epidemiology; MMAT, mixed methods appraisal tool.

## Data Availability

Not applicable.

## Supplementary Materials

The following supporting information can be downloaded at: https://www.mdpi.com/article/10.3390/jcm14217833/s1, Supplementary Materials File S1: PRISMA 2020 checklist (Table S1); Supplementary Materials File S2: Search strings used for each electronic database (Table S2), Eligibility criteria (Table S3), summary of quality assessments for the following tools: MMAT appraisal tool (Table S4a), JBI appraisal checklist (Table S4b), the STROBE checklist for cross-sectional studies (Table S5a), and the STROBE checklist for cohort studies (Table S5b).

## Abbreviations

The following abbreviations are used in this manuscript:

LTCFs	Long Term Care Facilities
NHs	Nursing Homes
LNs	Licensed Nurses
NAs	Nursing Assistants
HPRD	Hours per Resident per Day
NSAIDs	Nonsteroidal Anti-Inflammatory Drugs
PRN	Pro Re Nata (as needed)
PICOST	Participants, Interventions, Comparators, Outcomes, Study design, and Timing
PRISMA	Preferred Reporting Items for Systematic Reviews and Meta-Analyses
MMAT	Mixed-Methods Appraisal Tool
JBI	Joanna Briggs Institute
STROBE	Strengthening the Reporting of Observational Studies in Epidemiology
MeSH	Medical Subject Headings
CINAHL	Cumulative Index to Nursing & Allied Health Literature
WHO	World Health Organization
USA	United States of America
