# Supplementary material for: Factors Associated with the Variation in Drug Prescription of Analgesics in Long-Term Care Facilities: A Systematic Review
Source: J Clin Med. 2025 Nov 4;14(21):7833. doi: 10.3390/jcm14217833 (PMC12610536; doi:10.3390/jcm14217833)
Supplement: Supplementary file 1 [file jcm-14-07833-s001.zip › Supplementary File S2.pdf]

Table S2: Search strings based on the Database

Data access on 17/03/2024 on Cochrane Library

ID No.	Search
#1	MeSH descriptor: [Nursing Homes] explode all trees
#2	MeSH descriptor: [Long-Term Care] explode all trees
#3	MeSH descriptor: [Assisted Living Facilities] explode all trees
#4	#1 or #2 or #3
#5	((nursing home OR long-term care facilit* OR long term care facilit* OR skilled nursing facilit* OR residential care facilit* OR convalescent home OR elder care facilit* OR assisted living facilit* OR elder care facilit*)):ti,ab,kw (Word variations have been searched)
#6	#4 or #5
#7	MeSH descriptor: [Drug Prescriptions] explode all trees
#8	((drug prescription OR pharmaceutical prescription OR medication prescription OR therapeutic regimen OR medication regimen OR prescribing behavior* OR prescribing behaviour* OR prescribed drug use* OR pharmaceutical treatment)):ti,ab,kw (Word variations have been searched)
#9	#7 or #8
#10	((variability OR variabilit* OR compar* OR comparativ* OR differenc* OR disparit*)):ti,ab,kw (Word variations have been searched)
#11	(paracetamol OR tylenol):ti,ab,kw (Word variations have been searched)
#12	(ibuprofen OR advil):ti,ab,kw (Word variations have been searched)
#13	("acetaminophen"):ti,ab,kw (Word variations have been searched)
#14	MeSH descriptor: [Analgesics] explode all trees
#15	MeSH descriptor: [Anti-Inflammatory Agents] explode all trees
#16	MeSH descriptor: [Pain Management] explode all trees
#17	(analgesi* OR analgesic agent OR pain management OR painkiller* OR analgesic OR anti-inflammatory agents OR anti inflammatory agents OR nonsteroidal anti-Inflammatory OR nonsteroidal anti-inflammatory OR non-steroidal anti-inflammatory OR anti-inflammatory analgesics OR corticosteroids OR antipyretics OR antifebrile agents OR aspirin OR metamizole OR diclofenac OR naproxen OR indomethacin OR celecoxib OR diflunisal OR flurbiprofen OR ketoprofen OR ketorolac OR mefenamic acid OR meloxicam OR sulindac OR suprofen OR opioid* OR opiat* OR narcotic* OR codeine OR hydrocodone OR vicodin OR oxycodone OR tramadol OR morphine OR methadone OR meperidine OR fentanyl OR buprenorphine OR nalorphine OR nalbuphine OR naloxone OR naltrexone):ti,ab,kw (Word variations have been searched)
#18	#14 or #15 or #16 or #17
#19	#11 or #12 or #13 or #18
#20	#6 and #9 and #10 and #19

Data access on 01/01/2024 on PubMed

No	Searches

1	exp Nursing Homes/
2	exp Homes for the Aged/
3	exp Long-Term Care/
4	1 or 2 or 3
5	((nursing home OR long-term care facilit* OR long term care facilit* OR skilled nursing facilit* OR residential care facilit* OR convalescent home OR elder care facilit* OR assisted living facilit* OR elder care facilit*)).mp.
6	4 or 5
7	exp Drug Prescription/
8	((drug prescription OR pharmaceutical prescription OR medication prescription OR therapeutic<br>regimen OR medication regimen OR prescribing behavior* OR prescribing behaviour* OR prescribed<br>drug use* OR pharmaceutical treatment)).mp
9	7 or 8
10	((variability OR variabilit* OR compar* OR comparativ* OR differenc* OR disparit*)).mp
11	(Ibuprofen OR advil). mp. [mp=title, abstract, original title, name of substance word, subject heading word, keyword heading word, protocol supplementary concept word, rare disease supplementary concept word, unique identifier]
12	(acetaminophen OR paracetamol). mp. [mp=title, abstract, original title, name of substance word, subject heading word, keyword heading word, protocol supplementary concept word, rare disease supplementary concept word, unique identifier]
13	(tylenol OR metamizole). mp. [mp=title, abstract, original title, name of substance word, subject heading word, keyword heading word, protocol supplementary concept word, rare disease supplementary concept word, unique identifier]
14	(diclofenac OR naproxen). mp. [mp=title, abstract, original title, name of substance word, subject heading word, keyword heading word, protocol supplementary concept word, rare disease supplementary concept word, unique identifier]
15	(indomethacin). mp. [mp=title, abstract, original title, name of substance word, subject heading word, keyword heading word, protocol supplementary concept word, rare disease supplementary concept word, unique identifier]
16	(celecoxib). mp. [mp=title, abstract, original title, name of substance word, subject heading word, keyword heading word, protocol supplementary concept word, rare disease supplementary concept word, unique identifier]
17	(diflunisal). mp. [mp=title, abstract, original title, name of substance word, subject heading word, keyword heading word, protocol supplementary concept word, rare disease supplementary concept word, unique identifier]
18	(flurbiprofen). mp. [mp=title, abstract, original title, name of substance word, subject heading word, keyword heading word, protocol supplementary concept word, rare disease supplementary concept word, unique identifier]
19	(ketoprofen). mp. [mp=title, abstract, original title, name of substance word, subject heading word, keyword heading word, protocol supplementary concept word, rare disease supplementary concept word, unique identifier]
20	(ketorolac). mp. [mp=title, abstract, original title, name of substance word, subject heading word, keyword heading word, protocol supplementary concept word, rare disease supplementary concept word, unique identifier]
21	(mefenamic acid). mp. [mp=title, abstract, original title, name of substance word, subject heading word, keyword heading word, protocol supplementary concept word, rare disease supplementary concept word, unique identifier]

22	(meloxicam). mp. [mp=title, abstract, original title, name of substance word, subject heading word, keyword heading word, protocol supplementary concept word, rare disease supplementary concept word, unique identifier]
23	(sulindac). mp. [mp=title, abstract, original title, name of substance word, subject heading word, keyword heading word, protocol supplementary concept word, rare disease supplementary concept word, unique identifier]
24	(suprofen). mp. [mp=title, abstract, original title, name of substance word, subject heading word, keyword heading word, protocol supplementary concept word, rare disease supplementary concept word, unique identifier]
25	(opioid* OR opiat*). mp. [mp=title, abstract, original title, name of substance word, subject heading word, keyword heading word, protocol supplementary concept word, rare disease supplementary concept word, unique identifier]
26	narcotic*. mp. [mp=title, abstract, original title, name of substance word, subject heading word, keyword heading word, protocol supplementary concept word, rare disease supplementary concept word, unique identifier]
27	(hydrocodone OR Vicodin). mp. [mp=title, abstract, original title, name of substance word, subject heading word, keyword heading word, protocol supplementary concept word, rare disease supplementary concept word, unique identifier]
28	(Oxycodone). mp. [mp=title, abstract, original title, name of substance word, subject heading word, keyword heading word, protocol supplementary concept word, rare disease supplementary concept word, unique identifier]
29	(Tramadol). mp. [mp=title, abstract, original title, name of substance word, subject heading word, keyword heading word, protocol supplementary concept word, rare disease supplementary concept word, unique identifier]
30	(Morphine). mp. [mp=title, abstract, original title, name of substance word, subject heading word, keyword heading word, protocol supplementary concept word, rare disease supplementary concept word, unique identifier]
31	(Methadone). mp. [mp=title, abstract, original title, name of substance word, subject heading word, keyword heading word, protocol supplementary concept word, rare disease supplementary concept word, unique identifier]
32	(Meperidine). mp. [mp=title, abstract, original title, name of substance word, subject heading word, keyword heading word, protocol supplementary concept word, rare disease supplementary concept word, unique identifier]
33	(Fentanyl). mp. [mp=title, abstract, original title, name of substance word, subject heading word, keyword heading word, protocol supplementary concept word, rare disease supplementary concept word, unique identifier]
34	(Buprenorphine). mp. [mp=title, abstract, original title, name of substance word, subject heading word, keyword heading word, protocol supplementary concept word, rare disease supplementary concept word, unique identifier]
35	(Nalorphine). mp. [mp=title, abstract, original title, name of substance word, subject heading word, keyword heading word, protocol supplementary concept word, rare disease supplementary concept word, unique identifier]
36	(Nalbuphine). mp. [mp=title, abstract, original title, name of substance word, subject heading word, keyword heading word, protocol supplementary concept word, rare disease supplementary concept word, unique identifier]
37	exp Analgesic Agents/
38	(analgesi* OR analgesic agent OR pain management OR painkiller* OR analgesic OR anti-inflammatory agents OR anti inflammatory agents OR nonsteroidal anti Inflammatory OR nonsteroidal anti-inflammatory OR non-steroidal anti-inflammatory OR corticosteroids).mp. [mp=title, abstract, original title, name of substance word, subject heading word, keyword

	heading word, protocol supplementary concept word, rare disease supplementary concept word, unique identifier]
39	(antipyretics OR antifebrile agents). mp. [mp=title, abstract, original title, name of substance word, subject heading word, keyword heading word, protocol supplementary concept word, rare disease supplementary concept word, unique identifier]
40	38 or 30 or 40
41	11 or 12 or 13 or 14 or 15 or 16 or 17 or 18 or 19 or 20 or 21 or 22 or 23 or 24 or 25 or 26 or 27 or 28 or 29 or 30 or 31 or 32 or 33 or 34 or 35 or 36 or 37 or 38 or 39 or 40
42	6 and 9 and 10 and 41
43	Limit to last 11 years
44	Limit to English

Data access on 01/03/2024 on Scopus

Serial No.	Searches
1	TI,AB.KW(("nursing home" OR "long-term care facilit*" OR "long term care facilit*" OR "skilled nursing facilit*" OR "residential care facilit*" OR "convalescent home" OR "elder care facilit*" OR "assisted living facilit*" OR "elder care facilit*"))
2	TI,AB.KW ("variability" or "variabilit*" or "compar*" or "comparativ*" or "differenc*" or "disparit*")
3	TI,AB.KW ("drug prescription" or "pharmaceutical prescription" or "medication prescription" or "therapeutic regimen" or "medication regimen" or "prescribing behavior*" or "prescribing behaviour*" or "prescribed drug use*" or "pharmaceutical treatment")
4	(analgesi* OR "analgesic agent" OR "pain management" OR painkiller* OR analgesic OR "anti-inflammatory agents" OR "anti inflammatory agents" OR "nonsteroidal anti-Inflammatory" OR "nonsteroidal anti-inflammatory" OR "non-steroidal anti-inflammatory" OR "anti-inflammatory analgesics" OR corticosteroids OR antipyretics OR "antifebrile agents" OR aspirin OR ibuprofen OR advil OR acetaminophen OR paracetamol OR tylenol OR metamizole OR diclofenac OR naproxen OR indomethacin OR celecoxib OR diflunisal OR flurbiprofen OR ketoprofen OR ketorolac OR "mefenamic acid" OR meloxicam OR sulindac OR suprofen OR opioid* OR opiat* OR narcotic* OR "partial opioid agonist*" OR codeine OR hydrocodone OR vicodin OR oxycodone OR tramadol OR morphine OR methadone OR meperidine OR fentanyl OR buprenorphine OR nalorphine OR nalbuphine OR naloxone OR naltrexone)
5	(elder* OR senior* OR geriatric* OR convalesce* OR "older adult" OR "old age" OR "aged population")
6	(#1 AND #2 AND #3 AND #4 AND #5)
7	Limit to English and year 2013-2024

Data access on 17/03/2023

**Serial number**	**Searches**
1	(MH "Nursing Homes+") OR (MH " Long-Term Care ")
2	((MH "Nursing Homes" OR AB "long-term care facilit*" OR "long term care facilit*" OR AB "skilled nursing facilit*" OR "residential care facilit*" OR "Convalescent Care" OR "elder care facilit*" OR "assisted living facilit*"))
3	S1 OR S2

4	((variability OR variabilit* OR compar* OR comparativ* OR differenc* OR disparit*))
5	(("Drug Prescriptions" OR "pharmaceutical prescription" OR AB "medication prescription" OR "therapeutic regimen*" OR "medication regimen*" OR AB "prescribing behavior*" OR "prescribing behaviour*" OR AB "prescribed drug use*"OR "pharmaceutical treatment*"))
6	((analgesi* OR "analgesic agent"" OR ""pain management"" OR painkiller* OR analgesic OR "anti-inflammatory agents" OR "anti inflammatory agents" OR ""nonsteroidal antiInflammatory"" OR ""nonsteroidal antiinflammatory"" OR ""nonsteroidal antiinflammatory"" OR ""antiinflammatory analgesics"" OR corticosteroids OR antipyretics OR antifebrile agents OR aspirin OR ibuprofen OR advil OR acetaminophen OR paracetamol OR tylenol OR metamizole OR diclofenac OR naproxen OR indomethacin OR celecoxib OR diflunisal OR flurbiprofen OR ketoprofen OR ketorolac OR mefenamic acid OR meloxicam OR sulindac OR suprofen OR opioid* OR opiat* OR narcotic* OR "partial opioid agonist*" OR codeine OR hydrocodone OR vicodin OR oxycodone OR tramadol OR morphine OR methadone OR meperidine OR fentanyl OR buprenorphine OR nalorphine OR nalbuphine OR naloxone OR naltrexone))
7	(MH "Analgesic Agents") OR (MH Analgesics+")
8	S6 OR S7
9	S3 AND S4 AND S5 AND S8
10	LIMITED TO 2013-2024 & ENGLISH

Table S3: Eligibility criteria for analgesic drugs

Parameter	Inclusion Criteria	Exclusion Criteria
Population	Average age ≥ 60 years- LTCF residents prescribed analgesics for pain or cognitive impairment	Terminally ill patients receiving special care- Patients in hospice care
Intervention	Factors associated with variability in analgesic prescriptions in LTCFs	Factors associated with patients in hospice/inpatient care
Comparison	Not applicable	Not applicable
Outcomes	Variability in prescription and/or use of analgesics	Other outcomes not related to analgesic prescription variability
Study Design	Experimental, observational, and quasi-experimental studies based on primary data	Study protocols or those without original data

Timing	Published in English- Within the past 10 years (from January 2013 to January 2024)	Not applicable

Table S4a. Summary of quality assessments using MMAT appraisal tool.

Sections	*Question Number*	*Response*
1. Screening Questions	*Q1*	*YES*
	Q2	*YES*
		Eligibility passed
1. Qualitative	*Q1.1*	*YES*
	Q1.2	*YES*
	Q1.3	*YES*
	Q1.4	*YES*
	Q1.5	*YES*
		*5/5 = 100% or ***** (5 stars)*
2. Quantitative RCTs	*Q2.1*	*Not applicable*
	Q2.2	*Not applicable*
	Q2.3	*Not applicable*
	Q2.4	*Not applicable*
	Q2.5	*Not applicable*
3. Quantitative Nonrandomized	*Q3.1*	*YES*
	Q3.2	*YES*
	Q3.3	*YES*
	Q3.4	*NO*
	Q3.5	*NO*
		*3/5 = 60% or *** (3 stars)*
4. Quantitative Descriptive	*Q4.1*	*YES*
	Q4.2	*YES*
	Q4.3	*YES*
	Q4.4	*YES*
	Q4.5	*YES*
		*5/5 = 100% or ***** (5 stars)*
5. Mixed Methods	*Q5.1*	*YES*
	Q5.2	*YES*
	Q5.3	*YES*
	Q5.4	*NO*
	Q5.5	*NO*
		*3/5 = 60% or *** (3 stars)*
Total score		*QUAL = 5*<br>*QUAN = 3 (nonrandomized)*<br>*MM = 3*<br>*Overall = 3 (60%) or *** (3 stars)*

Reference [20]

MMAT Criteria to be scored for mixed method study):

1. ***Screening questions (for all types):***
   Q1. Are there clear research questions?; Q2. Do the collected data allow to address the research questions?.;

2. ***Qualitative:*** *Q2.1 Is the qualitative approach appropriate to answer the research question?.; Q2.2. Are the qualitative data collection methods adequate to address the research question?; Q2.3 Are the findings adequately derived from the data?.; Q2.4. Is the interpretation of results sufficiently substantiated by data?; Q2.5.Is there*

coherence between qualitative data sources, collection, analysis and interpretation?

3. ***Quantitative nonrandomized:*** *Q3.1 Are the participants representative of the target population?.; Q3.2. Are measurements appropriate regarding both the outcome and intervention (or exposure)?; Q3.3. Are there complete outcome data?; Q3.4 Are the confounders accounted for in the design and analysis?.; Q3.5. During the study period, is the intervention administered (or exposure occurred) as intended?*

4. ***Quantitative descriptive****: Q4.1. Is the sampling strategy relevant to address the research question?;. Q4.2. . Is the sample representative of the target population?;.Q4.3. Are the measurements appropriate?.; Q4.4. Is the risk of nonresponse bias low?.; Q4.5. Is the statistical analysis appropriate to answer the research question?*

5. ***Mixed methods:*** *Q5.1. Is there an adequate rationale for using a mixed methods design to address the research question?.; Q5.2. Are the different components of the study effectively integrated to answer the research question?.; Q5.3. Are the outputs of the integration of qualitative and quantitative components adequately interpreted?.; Q5.4. Are divergences and inconsistencies between quantitative and qualitative results adequately addressed?.; Q5.5. Do the different components of the study adhere to the quality criteria of each tradition of the methods involved?*

Reporting the results of the MMAT according to the 2018 version.
For each retained study, an overall quality score using the MMAT (Mixed Methods Appraisal Tool) may not always be the most informative option compared to a descriptive summary based on the specific MMAT criteria. However, if a score is to be calculated, it can be presented using simple descriptors such as stars (*) or percentages (%):

1. 5 stars (*): 100% of quality criteria met
2. 4 stars (****): 80% of quality criteria met
3. 3 stars (*):** 60% of quality criteria met
4. 2 stars ():** 40% of quality criteria met
5. 1 star (*): 20% of quality criteria met

For mixed methods studies, since they include 15 criteria across three domains (qualitative, quantitative, and mixed methods), the overall quality score is determined by the lowest score among the three components. This is based on the principle that the quality of a mixed methods study cannot exceed the quality of its weakest part.

1. The score is 20% (*) if any of QUAL, QUAN, or MM scores = 1
2. The score is 40% ()** if any score = 2
3. The score is 60% (*)** if any score = 3
4. The score is 80% (****) if all three components score 4
5. The score is 100% (*) if any of the three components scores 5

Where:

1. QUAL = score of the qualitative component
2. QUAN = score of the quantitative component
3. MM = score of the mixed methods component

Table S4b. Summary of quality assessments using JBI appraisal checklist.

Study	Study design	Q1	Q2	Q3	Q4	Q5	Q6	Q7	Q8	Q9	Q10	Raw score and %
Corazzini et al., 2013	Qualitative	1	1	1	1	1	0	0	1	0	1	7/10=70%

Reference [15]

***JBI Criteria to be scored for Qualitative study**: Q1. Is there congruity between the stated philosophical perspective and the research methodology; Q2. Is there congruity between the research methodology and the research question or objectives; Q3. Is there congruity between the research methodology and the methods used to collect data; Q4. Is there congruity between the research methodology and the representation and analysis of data; Q5. Is there congruity between the research methodology and the interpretation of results; Q6. Is there a statement locating the researcher culturally or theoretically; Q7. Is the influence of the researcher on the research, and vice- versa, addressed; Q8. Are participants, and their voices, adequately represented; Q9. Is the research ethical according to current criteria or, for recent studies, and is there evidence of ethical approval by an appropriate body; Q10. Do the conclusions drawn in the research report flow from the analysis, or interpretation, of the data.*

1 = Yes, 0 = No, and U = Unclear.

***Abbreviations:** 1 = Yes; 0 = No; U = Unclear; NA = Not Applicable; JBI: Joanna Briggs Institute*

Criteria used to rank the risk of bias

i) ≤49% = high risk of Bias

ii) 50% and 69% = Medium risk of Bias

iii) Above 70% = low risk of Bias

Table S5a. Summary of quality assessments using STROBE checklist for Cross-sectional studies

Study	Q1	Q2	Q3	Q4	Q5	Q6	Q7	Q8	Q9	Q10	Q11	Q12	Q13	Q14	Q15	Q16	Q17	Q18	Q19	Q20	Q21	Q22	Total
Fain et al 2013	2	1	1	1	1	1	1	1	1	0	1	3	2	2	1	2	1	1	1	1	1	1	27
baretto et al 2013	2	1	1	1	1	1	1	1	1	1	1	3	1	1	1	1	1	1	1	1	1	1	25
Jensen Dahem 2015	2	1	1	1	1	1	1	1	1	1	1	2	3	1	1	1	1	1	1	1	1	1	26
Lacono 2022	1	1	1	1	1	1	1	1	1	0	1	1	0	0	1	1	1	1	1	1	1	1	19
lapane 2019	1	1	1	1	1	1	1	1	0	0	0	1	1	1	1	2	0	1	0	1	1	1	18
lukas 2013	1	1	1	1	1	1	1	1	1	1	1	2	2	1	1	1	1	1	1	1	1	0	23
Martens 2018	1	1	1	1	1	1	1	0	0	0	0	1	0	1	1	1	0	1	1	0,25	0	0	13,25
sandvik 2016	1	1	0	1	1	1	1	1	0	0	0	1	0	1	1	2	1	1	0	0	0	1	15
Vael 2015	2	1	1	1	1	1	1	1	0	0	0	1	2	1	1	2	0,5	1	1	0,5	1	0	20

Reference [16]

Criteria used to rank the risk of bias

i) 0-14 score = high risk of Bias

ii) 15-25 = Medium risk of Bias

iii) 26-33 = low risk of Bias

Table S5b. Summary of quality assessments using STROBE checklist for Cohort studies

Study	Q1	Q2	Q3	Q4	Q5	Q6	Q7	Q8	Q9	Q10	Q11	Q12	Q13	Q14	Q15	Q16	Q17	Q18	Q19	Q20	Q21	Q22	Total
Mehta 2021	1	1	1	1	1	1	1	1	0	0	0	1	1	1	1	1	0	1	1	0	1	1	17
Ranchon 2023	2	1	1	1	1	1	1	1	0	0	1	1	0,5	1	1	2	0	1	1	1	1	1	20,5

Reference [16]

Criteria used to rank the risk of bias

i) 0-14 score = high risk of Bias

ii) 15-25 = Medium risk of Bias

iii) 26-33 = low risk of Bias
